# Skeletal Muscle Adaptations and Performance Outcomes Following a Step and Exponential Taper in Strength Athletes

**DOI:** 10.3389/fphys.2021.735932

**Published:** 2021-10-21

**Authors:** S. Kyle Travis, Kevin A. Zwetsloot, Iñigo Mujika, Michael H. Stone, Caleb D. Bazyler

**Affiliations:** ^1^Exercise and Sport Sciences Laboratory, Department of Sport, Exercise, Recreation, and Kinesiology, East Tennessee State University, Johnson City, TN, United States; ^2^Department of Rehabilitative Sciences, East Tennessee State University, Johnson City, TN, United States; ^3^Integrative Muscle Physiology Laboratory, Department of Health and Exercise Science, Appalachian State University, Boone, NC, United States; ^4^Department of Biology, Appalachian State University, Boone, NC, United States; ^5^Department of Physiology, Faculty of Medicine and Nursing, University of the Basque Country, Leioa, Spain; ^6^Exercise Science Laboratory, School of Kinesiology, Faculty of Medicine, Universidad Finis Terrae, Santiago, Chile

**Keywords:** powerlifting, muscle biopsy, fiber typing, gene expression, mRNA, resistance training, myosin heavy chain, maximal strength

## Abstract

Before major athletic events, a taper is often prescribed to facilitate recovery and enhance performance. However, it is unknown which taper model is most effective for peaking maximal strength and positively augmenting skeletal muscle. Thus, the purpose of this study was to compare performance outcomes and skeletal muscle adaptations following a step vs. an exponential taper in strength athletes. Sixteen powerlifters (24.0 ± 4.0 years, 174.4 ± 8.2 cm, 89.8 ± 21.4 kg) participated in a 6-week training program aimed at peaking maximal strength on back squat [initial 1-repetition-maximum (1RM): 174.7 ± 33.4 kg], bench press (118.5 ± 29.9 kg), and deadlift (189.9 ± 41.2 kg). Powerlifters were matched based on relative maximal strength, and randomly assigned to either (a) 1-week overreach and 1-week step taper or (b) 1-week overreach and 3-week exponential taper. Athletes were tested pre- and post-training on measures of body composition, jumping performance, isometric squat, and 1RM. Whole muscle size was assessed at the proximal, middle, and distal vastus lateralis using ultrasonography and microbiopsies at the middle vastus lateralis site. Muscle samples (*n* = 15) were analyzed for fiber size, fiber type [myosin-heavy chain (MHC)-I, -IIA, -IIX, hybrid-I/IIA] using whole muscle immunohistochemistry and single fiber dot blots, gene expression, and microRNA abundance. There were significant main time effects for 1RM squat (*p* < 0.001), bench press (*p* < 0.001), and deadlift, (*p* = 0.024), powerlifting total (*p* < 0.001), Wilks Score (*p* < 0.001), squat jump peak-power scaled to body mass (*p* = 0.001), body mass (*p* = 0.005), fat mass (*p* = 0.002), and fat mass index (*p* = 0.002). There were significant main time effects for medial whole muscle cross-sectional area (mCSA) (*p* = 0.006) and averaged sites (*p* < 0.001). There was also a significant interaction for MHC-IIA fiber cross-sectional area (fCSA) (*p* = 0.014) with *post hoc* comparisons revealing increases following the step-taper only (*p* = 0.002). There were significant main time effects for single-fiber MHC-I% (*p* = 0.015) and MHC-IIA% (*p* = 0.033), as well as for MyoD (*p* = 0.002), MyoG (*p* = 0.037), and miR-499a (*p* = 0.033). Overall, increases in whole mCSA, fCSA, MHC-IIA fCSA, and MHC transitions appeared to favor the step taper group. An overreach followed by a step taper appears to produce a myocellular environment that enhances skeletal muscle adaptations, whereas an exponential taper may favor neuromuscular performance.

## Introduction

Before major competitions, a taper is often prescribed as the final stage of training aimed at decreasing physiological and psychological fatigue to achieve optimal preparedness (Mujika and Padilla, 2003; Travis et al., 2020c). A taper is typically constructed via reducing the amount of training, primarily through decreasing overall training volume-load and manipulating intensity over 1–4 weeks (Mujika and Padilla, 2003; Pritchard et al., 2015; Travis et al., 2020c). The manner in which work is reduced can be accomplished using different taper models including step, linear, and exponential with fast- or slow-decay (Mujika and Padilla, 2003). While the majority of the tapering literature has focused on endurance sport performances, the current literature for tapering and peaking maximal strength is scant.

The evidence for tapering and peaking maximal strength primarily consists of observational (Bazyler et al., 2017, 2018; Travis et al., 2020b) and qualitative research (Pritchard et al., 2016, 2020; Grgic and Mikulic, 2017; Winwood et al., 2018). To date, only two studies have experimentally compared tapering strategies aimed at improving maximal strength: (a) +5% vs. −10% intensity manipulation with a ∼70% volume-load reduction using a step taper (Pritchard et al., 2019), and (b) a step vs. an exponential taper with a ∼54% volume-load reduction while maintaining intensity (Seppänen and Häkkinen, 2020). In summary, these data suggest that a higher intensity taper and an exponential taper over 2-weeks may produce favorable outcomes. However, based on a recent systematic review, the optimal tapering duration for peaking maximal strength may be ≤2 weeks where volume-load is reduced by half while maintaining or reducing intensity (Travis et al., 2020c). Interestingly, survey results from 364 United States and Canadian powerlifters revealed lifters most frequently used a 7–10 day step taper with a 41–50% reduction in volume-load, but varied intensity manipulation (−30 to +10%) (Travis et al., 2021). Tapering for maximal strength appears to be more sensitive to volume-load reductions and possibly the duration of a pre-competition taper, rather than intensity manipulations. Nonetheless, the manner in which volume-load is reduced (e.g., step-fashion, exponentially decayed) over a 1 or 3-week duration with the aim of peaking maximal strength requires further examination.

According to Luden et al. (2010), the majority of tapering literature has focused on targeting taper-induced physiological adaptations relative to cardiovascular, metabolic, and neuromuscular parameters in an attempt to identify physiological factors supporting the ergogenic effect of tapering. However, these aforementioned parameters do not appear to adequately explain enhanced performance adaptations and these characteristics relatively remain unchanged with endurance athletes. Conversely, skeletal muscle size, force, and power characteristics appear to be more sensitive to tapered training with endurance and strength athletes, which could likely potentiate positive taper-induced training outcomes (Murach et al., 2014). Unfortunately, skeletal muscle adaptations at the whole muscle and single fiber levels as a result of tapering are poorly understood and should be considered per the relationships between altered training volume and muscle size (Haun et al., 2019; Travis et al., 2020a). For instance, if the taper duration is too long, negative skeletal muscle adaptations could provide evidence for avoiding long duration tapers (e.g., 3-week exponential taper) for strength athletes due to insufficient stimuli. However, skeletal muscle adaptations are often associated with interpretations that are speculative due to measurement limitations such as ultrasonography. Thus, additional assumptions are made regarding deeper levels of skeletal muscle constituents (e.g., muscle fiber size, myosin, and actin protein concentrations), which are also poorly understood and require further investigation.

Studies with strength athletes have demonstrated whole muscle cross-sectional area (mCSA) is maintained or slightly decreased during or after a taper possibly due to sustained reductions in training volume (Häkkinen et al., 1991; Zaras et al., 2014, 2016; Bazyler et al., 2017, 2018; Suarez et al., 2019; Seppänen and Häkkinen, 2020; Travis et al., 2020b). This is understandable given the direct relationship between training volume and muscle size (Schoenfeld et al., 2014; Haun et al., 2019). While performances can vary as a result of whole muscle maintenance or loss, it is unclear what underlying cellular or molecular changes are taking place at the muscle fiber level with strength-based tapers. Skeletal muscle is highly plastic, which is evidenced by changes in muscle fiber composition in response to different training stimuli (D’Souza et al., 2018; Travis et al., 2020a). Specifically, tapering has been shown to favor myosin-heavy chain (MHC)-IIA isoforms by increasing fiber cross-sectional area (fCSA), peak force, and power output (Trappe et al., 2000b; Neary et al., 2003; Luden et al., 2010; Murach et al., 2014), albeit in endurance athletes. Considering MHC-IIA muscle fibers are the most abundant and largest fibers in powerlifters (Prince et al., 1976; MacDougall et al., 1982; Tesch et al., 1984; Kadi et al., 1999; Fry et al., 2003; Eriksson et al., 2005; Machek et al., 2020), creating a cellular environment that enhances IIA fiber content may be warranted. Unfortunately, it is unknown whether similar muscular adaptations occur in strength athletes following a taper. Further, additional myocellular constituents (e.g., sarcoplasm, gene expression, and protein abundance) driving whole muscle and single fiber adaptations in strength athletes during a taper are still unclear.

When assessing taper-induced MHC isoform shifts, there are concurrent MHC isogenes [e.g., myosin-heavy chain 7 (MYH7), myosin-heavy chain 2 (MYH2), myosin-heavy chain 1 (MYH1)] (McDermott and Bonen, 1991) that encode different types of molecular motors (Goldspink, 2002). Over a decade ago, evidence was provided to support the mechanisms underlying skeletal muscle regulation which was linked to vast arrays of muscle-specific genes encoding for proteins that required specialized functions for contractile apparatuses, enzymes, receptors, and ion channels (Goldspink, 2002; van Rooij et al., 2008). Downstream from gene expression, collections of muscle-specific microRNAs (miR) (i.e., MyomiR) (McCarthy, 2011) mediated by cell proliferation, differentiation, contractility, and stress responses were identified. miR inhibits translation and promotes messenger RNA (mRNA) degradation. Thus, the interactions between genes and miR are vital for understanding molecular mechanisms that influence skeletal muscle adaptations.

Prior work by D’Souza et al. (2017) characterized powerlifters’ muscle phenotype via gene and miR expression suggesting that powerlifters possess unique expression profiles, compared to other populations. However, only one study has attempted to assess these molecular markers during a training protocol with powerlifters, albeit aimed at enhancing muscle hypertrophy (Bjørnsen et al., 2019a). Contrary to the notion that exercise gene response and adaptative potential are attenuated as training status improves, findings by Murach et al. (2014) suggest transcriptional flexibility in MHC-I and -IIA at the gene level can be observed after tapering in collegiate distance runners. Nonetheless, changes in gene and miR expression have not been studied in strength athletes following a taper. Given the influence of muscular adaptations on maximal strength outcomes (MacDougall et al., 1982; Brechue and Abe, 2002; Schoenfeld et al., 2014; Abe et al., 2018a,b), genes mediating muscle phenotype [i.e., SRY-box transcription factor 6 (SOX6), myosin-heavy gene 7 (MYH7), myosin-heavy gene 2 (MYH2), and myosin-heavy gene 1 (MYH1)], and regulating myogenesis [i.e., protein Pax7 (PAX7), Myostatin (MSTN), myogenic differentiation 1 (MyoD), and myogenin (MyoG)] may elucidate tapering-induced muscular adaptations at the molecular level (McCarthy, 2011; D’Souza et al., 2017; Bjørnsen et al., 2019a). In addition to gene expression, examining muscle-specific miRs highly expressed in powerlifters (i.e., miR-133a, -206, -486, and -499a), and indicative of catabolic gene inhibition (i.e., miR-23 and miR-451) may enhance our understanding of the gene-miR interactions with subsequent muscular adaptations following tapering. Whether taper models commonly used by strength athletes exhibit differences in gene expression and corresponding muscle phenotype remains to be investigated.

Currently, it is unclear which tapering model is most effective for peaking maximal strength and positively augmenting skeletal muscle. Additionally, beyond macroscopic assessments, molecular measurements are needed to further understand taper-induced adaptations. Thus, the purpose of this study was to compare performance outcomes and skeletal muscle adaptations following a 6-week peaking program using a step or exponential taper in strength athletes.

## Materials and Methods

### Ethical Approval and Participant Screening

A total of 16 powerlifters (14 males and 2 females; 24.2 ± 4.0 years; 174.4 ± 8.2 cm; 89.8 ± 21.4 kg) read and signed an informed consent document before beginning the study. This protocol was reviewed and approved by the East Tennessee State Institutional Review Board and complied with the Helsinki Declaration (approved protocol # 0191.15f). Inclusion criteria were as follows: (1) 18–35 years old; (2) free from injury/illness that would hinder participation; (3) regularly trained the powerlifting movements (i.e., back squat, bench press, and deadlift) over the last year; and (4) could demonstrate a 1-repetition-maximum (1RM) strength level ≥1.5× body mass on back squat and deadlift, and ≥1× body mass on bench press. Participants completed all testing and training sessions, and there were no dropouts.

### Experimental Design

**FIGURE 1:**
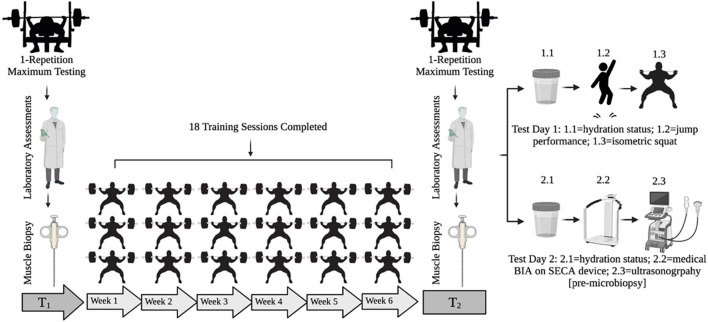
Testing timeline. T_1_ = pre-training testing week. T_2_ = post-taper testing week. BIA = bioelectrical impedance analysis.

An experimental design was used to compare a 1-week step vs. a 3-week exponential taper completed during a 6-week peaking program. All participants were familiarized with testing procedures over 4-weeks prior to beginning the study. Before any training, participants underwent a 1RM testing session where they were ranked based on calculated Wilks Score (i.e., coefficient used to compare relative strength across differing body mass and between sex) (Vanderburgh and Batterham, 1999). Matched pairs were randomly assigned to either the step or exponential taper by an assistant unaffiliated with the study. All participants trained 3 days per week at the same time of day. At the end of each training session, participants provided session rating of perceived exertion (1–10) and duration (min), which were used to calculate weekly training monotony and strain (Borg, 1962; Pyne et al., 2000). Participants were instructed to arrive at the laboratory in a fully rested, hydrated state, refrain from training and stimulants, and complete a 48-h dietary log prior to the first testing session to be replicated prior to the second testing session. Participants completed two testing sessions: 1 week prior to any training (T_1_) and 1 week post-taper (T_2_) (Figure 1).

### Training Program

An experimental design was used to compare a 1-week step vs. a 3-week exponential taper completed during a 6-week peaking program. All participants were familiarized with testing procedures over 4-weeks prior to beginning the study. Before any training, participants underwent a 1RM testing session where they were ranked based on calculated Wilks Score (i.e., coefficient used to compare relative strength across differing body mass and between sex) (Vanderburgh and Batterham, 1999). Matched pairs were randomly assigned to either the step or exponential taper by an assistant unaffiliated with the study. All participants trained 3 days per week at the same time of day. At the end of each training session, participants provided session rating of perceived exertion (1–10) and duration (min), which were used to calculate weekly training monotony and strain (Borg, 1962; Pyne et al., 2000; Foster et al., 2001). Participants were instructed to arrive at the laboratory in a fully rested, hydrated state, refrain from training and stimulants, and complete a 48-h dietary log prior to the first testing session to be replicated prior to the second testing session. Participants completed two testing sessions: 1 week prior to any training (T_1_) and 1 week post-taper (T_2_) (Tables 1, 2).

**TABLE 1:** Testing timeline and exercise prescription.

Step Taper Group		Day 1	Day 2	Day 3
	*Testing (T_1_)*	*1RM testing session*	*Lab testing*	*Biopsy sampling*
	Week 1	DL, OHP, BBR, RF	BS, BP, PU, DP	BS, BP, PU, DP
	Week 2	DL, OHP, BBR, RF	BS, BP, PU, DP	BS, BP, PU, DP
	Week 3	BS, BP, DL, OHP	BS, BP, PU, DP	BS, BP, PR, CGBP
	Week 4	BS, BP, DL, OHP	BS + BS, BP + BP, PU, DP	BS + BS, BP + BP, PR
	^*^Week 5	BS, BP, DL, OHP	BS, BP, PU, DP	BS, BP, PR, CGBP
	^#^Week 6	BS + BS, BP + BP, DL + DL, OHP	BS + BS, BP + BP, PU, DP	BS + BS, BP + BP, PR
	*Testing (T_2_)*	*1RM testing session*	*Lab testing*	*Biopsy sampling*

**Exponential Taper Group**		**Day 1**	**Day 2**	**Day 3**

	*Testing (T_1_)*	*1RM testing session*	*Lab testing*	*Biopsy sampling*
	Week 1	DL, OHP, BBR, RF	BS, BP, PU, DP	BS, BP, PU, DP
	Week 2	DL, OHP, BBR, RF	BS, BP, PU, DP	BS, BP, PU, DP
	^*^Week 3	BS, BP, DL, OHP	BS, BP, PU, DP	BS, BP, PR, CGBP
	^#^Week 4	BS, BP, DL, OHP	BS, BP, PU, DP	BS, BP, PR, CGBP
	^##^Week 5	BS, BP, DL, OHP	BS + BS, BP + BP, PU, DP	BS + BS, BP + BP, PR
	^###^Week 6	BS + BS, BP + BP, DL + DL, OHP	BS + BS, BP + BP, PU, DP	BS + BS, BP + BP, PR
	*Testing (T_2_)*	*1RM testing session*	*Lab testing*	*Biopsy sampling*

*T_1_ = pre-training testing week; T_2_ = post-taper intervention testing week; DL = deadlift; OHP = over-head press; BBR = bent over barbell row; RF = rear fly; BS = back squat; BP = bench press; PU = pull-ups; DP = dips; PR = pendlay row; CGBP = close-grip bench press; BS + BS = back squat with downsets; BP + BP = bench press with downsets; DL + DL = deadlift with downsets.*

*Denotes planned overreach.

*^#^Denotes taper week 1.*

*^##^Denotes taper week 2.*

*^###^Denotes taper week 3.*

**TABLE 2:** Training program prescription for relative training intensity, sets, and repetitions.

Step Taper Group		Day 1		Day 2		Day 3	
		RI	Sets × Reps	RI	Sets × Reps	RI	Sets × Reps
	Week 1	82.5–87.5%	4 × 5	77.5–82.5%	4 × 5	80.0–85.0%	4 × 5
	Week 2	85.0–90.0%	4 × 5	82.5–87.5%	4 × 5	87.5–92.5%	4 × 5
	Week 3	87.5–92.5%	4 × 3	82.5–87.5%	4 × 5	85.0–90.0%	4 × 5
	Week 4	90.0–95.0%	4 × 3	80.0–85.0%	3 × 3 + 2 × 5; 3 × 5	82.5–87.5%	3 × 2 + 2 × 5; 3 × 5
	^*^Week 5	82.5–87.5%	7 × 3	77.5–82.5%	7 × 5	80.0–85.0%	7 × 5
	^#^Week 6	90.0–95.0%	1 × 1 + 3 × 2; 3 × 2	85.0–90.0%	3 × 2 + 2 × 5; 3 × 5	70.0–75.0%	3 × 2 + 2 × 5; 3 × 5

**Exponential Taper Group**		**Day 1**		**Day 2**		**Day 3**	
		**RI**	**Sets** × **Reps**	**RI**	**Sets** × **Reps**	**RI**	**Sets** × **Reps**

	Week 1	82.5–87.5%	4 × 5	77.5–82.5%	4 × 5	80.0–85.0%	4 × 5
	Week 2	85.0–90.0%	4 × 5	82.5–87.5%	4 × 5	87.5–92.5%	4 × 5
	*Week 3	82.5–87.5%	7 × 3	77.5–82.5%	7 × 5	80.0–85.0%	7 × 5
	^#^Week 4	87.5–92.5%	4 × 3	82.5–87.5%	4 × 5	85.0–90.0%	4 × 5
	^##^Week 5	90.0–95.0%	4 × 3	80.0–85.0%	3 × 3 + 2 × 5; 3 × 5	82.5–87.5%	3 × 2 + 2 × 5; 3 × 5
	^###^Week 6	90.0–95.0%	1 × 1 + 3 × 2; 3 × 2	85.0–90.0%	3 × 2 + 2 × 5; 3 × 5	70.0–75.0%	3 × 2 + 2 × 5; 3 × 5

*RI = relative training intensity of%1-repeitiom maximum.*

^*^Denotes planned overreach.

*^#^Denotes taper week 1.*

*^##^Denotes taper week 2.*

*^###^Denotes taper week 3.*

*For sets × reps such as 3 × 3 + 2 × 5, this indicates primary exercise work pre-scribed (3 × 3) followed by additional work (2 × 5) as described via exercise selection in Table 1. If a secondary assistance exercise is included in the session, the set and repetition scheme is followed by a semicolon (e.g., 3 × 3 + 2 × 5; 3 × 5). Refer to Table 1 to reference exercise prescription.*

### Laboratory Testing Procedures

#### Hydration Assessment

Hydration status was evaluated at the start of each laboratory assessment using a refractometer (ATAGO 4410 PAL-10S, Tokyo, Japan). If urine-specific gravity was ≥1.020, the participant was required to drink water for at least 20 min before hydration status was reassessed. Participants were not allowed to continue testing until urine-specific gravity reached <1.020.

#### Jump Performance Assessment

Participants performed a standardized dynamic warm-up to prepare for squat jumps (SJ). Unloaded SJs were performed on dual uniaxial force plates affixed side by side with a sampling frequency of 1,000 Hz (Rice Lake Weighing Systems, Rice Lake, WI, United States). The SJ was performed with a near weightless plastic pipe placed across the shoulders to eliminate arm swing. The tester instructed each participant to squat down to a 90° knee angle, which was confirmed with a handheld goniometer. Two warm-ups were completed at 50% and 75% maximal effort. When commanded, each participant was instructed to “step on the force plate,” receive the “ready position” and hold the 90° squat position until the force-time trace was stable for at least 2 s. The tester then shouted “3, 2, 1, jump!” and the participant executed a maximal effort jump. Using the live SJ jump-height (SJH) metric, at least two jumps were performed within a range of ≤2 cm. All jump trials were recorded and analyzed using a custom program (LabView, 2018, National Instruments Co., Austin, TX, United States). Variables of interest (SJH: interclass correlation coefficient (ICC) = 0.96, coefficient of variation (CV) = 2.55%; peak power allometrically scaled for body mass (PPa) (ICC = 0.96, CV = 2.08%) yielded repeated measurement values consistent with previous reports from our laboratory (Bazyler et al., 2018; Travis et al., 2020b).

#### Isometric Squat Assessment

Participants were positioned in a custom designed power rack that allows fixation of the bar at any height as described previously (Bazyler et al., 2015). Each participant’s bar height was determined by a 90° knee angle confirmed with a handheld goniometer referencing the greater trochanter, lateral epicondyle, and lateral malleolus for the appropriate isometric squat (ISQ) position. The same investigator measured bar height and knee angle, which was replicated at each testing session. Kinetic variables were measured on dual uniaxial force plates affixed side by side with a sampling frequency of 1,000 Hz. Prior to maximal testing, two warm-up trials were provided at 50 and 75% maximal effort. At least two maximal effort trials were administered. Additional trials were provided if isometric peak force (IPF) values were ≥100 N. The ISQ trials with the two highest IPF values were analyzed using a custom program (LabView, 2018, National Instruments Co., Austin, TX, United States). Test–retest reliability for ISQ IPF was nearly perfect (ICC = 0.99, CV = 1.97%).

#### One-Repetition-Maximum Assessment

Participants underwent two 1RM testing sessions at T1 and T2 in the form of mock competitions aimed at achieving a true 1RM. Both mock competitions were supervised and performed in accordance with USA Powerlifting (USAPL) rules while using validated 1RM and attempt selection procedures (Zourdos et al., 2016; USAPL and Administrators, 2019; Travis et al., 2020d). The primary investigator determined load increases for each attempt for all participants under the following conditions: (a) a rating of perceived exertion of 10 being recorded and the investigator determined that any load increase would not result in a successful attempt or the participant failing on any subsequent attempt thereafter or (b) a recorded rating of perceived exertion of 9 or 9.5 and then the participant failing on the subsequent attempt with a load increase of ≤2.5 kg.

### Skeletal Muscle Measurements

#### Body Composition Assessment

A medical body composition analyzer (SECA mBCA 515 v1.1 Hamburg, Germany) using bioelectrical impedance analysis (BIA) was used to determine body mass, fat mass (FM), fat free mass (FFM), fat mass index (FMI), fat free mass index (FFMI), total body water (TBW) [i.e., composed of extracellular water (ECW) and intracellular water], and skeletal muscle mass. Impedance was measured at frequencies ranging from 1 to 1,000 kHz (Peine et al., 2013). The measurement scanning sequence was performed segmentally in accordance with the user instruction manual in the following order: right arm, left arm, right leg, left leg, trunk, right body side, and left body side (Peine et al., 2013). Test–retest reliability was nearly perfect for all SECA variables with an ICC = 0.98 to 0.99 and CV = 1.76 to 3.41% (Bosy-Westphal et al., 2013, 2017; Peine et al., 2013; Jensen et al., 2019).

#### Ultrasonography

Anatomical mCSA of the right vastus lateralis was assessed using 2D ultrasonography (LOGIQ P6, General Electric Healthcare, Wauwatosa, WI, United States). A 7.5 MHz ultrasound probe was covered with water-soluble transmission gel to aid acoustic coupling. Depression of the skin was avoided while collecting the cross-sectional image. Sampling locations of interest were mCSA_1__/__3_ (proximal), mCSA_1__/__2_ (middle), and mCSA_2__/__3_ (distal) of femur length, which was determined by the distance between the greater trochanter and the lateral epicondyle of the femur. Each sub-region was measured, recorded, and marked with a permanent marker to guide biopsy procedures relative to the area of interest. Each participant laid on their left side in a recovery position with hips perpendicular to the examination table in the axial plane. The mean of three images from each sub-section was used for analysis. For analysis, mCSA was measured by tracing the inter-muscular interface around each muscle cross-sectional image using a secondary software (Image-J Fiji version 2.0.0-rc-68/1.52g, National Institutes of Health, Bethesda, MD, United States) (Schindelin et al., 2015). mCSA from individual sites (mCSA_1__/__3_, mCSA_1__/__2_, and mCSA_2__/__3_) and the mean of three sites (mCSA_*avg*_) were analyzed to assess changes in regional and whole muscle size, respectively. Test–retest reliability was nearly perfect (ICC: 0.99; CV = 0.98%) and agrees with previous reports published from the same technician (Travis et al., 2020b).

#### Muscle Biopsy

Following ultrasound scans, local anesthesia (1% xylocaine, Hospira, Inc., Lake Forest, IL, United States) was injected subcutaneously in the right vastus lateralis at the site corresponding to the mCSA_1/2_ femur length ultrasound marking. Muscle biopsy samples were obtained using a 14G × 9 cm biopsy instrument with a 13G × 3.9 cm co-axial introducer (MCXS1409LX SuperCore^TM^ Semi-Automatic Biopsy Instrument, Argon Medical Devices, Frisco, TX, United States). After placing the introducer needle, 5–6 passes were performed with the biopsy needle extracting approximately 15–20 mg of muscle tissue per pass. As a result, muscle samples totaled approximately 75–120 mg, which were separated from connective and adipose tissue. Approximately four fascicles were removed from the total muscle sample and mounted in duplicate on corks in tragacanth gum/optimal cutting temperature mixture (Thermo Fisher Scientific Inc., Waltham, MA, United States) and frozen in liquid N_2_-cooled isopentane for histological analyses. The remaining tissues were then weighed, equally divided into 3–4 CryoTube^®^ vials (Nunc^®^, Roskilde, Denmark), flash frozen in liquid nitrogen, and stored in −80°C for subsequent analyses. These procedures were implemented at T_1_ and T_2_.

#### Immunohistochemical Analysis

Cork mounted samples were cut into 10 μm cross-sections using a cryostat (Model Microm HM 505; Thermo Fisher Scientific Inc.) and mounted on positively charged microscope slides (Fisherbrand^TM^ Superfrost Plus; Fisher Scientific, Pittsburgh, PA, United States). Section quality and tissue integrity were assessed using hematoxylin and eosin staining. MHC fiber type immunofluorescent detection was performed using published methods with modifications for human skeletal muscle (Lawrence et al., 2021). Muscle sections were blocked with 10% normal goat serum in 1 × PBS and incubated overnight (12 h) in 4°C with a primary antibody (1°Ab) cocktail obtained from the Developmental Studies Hybridoma Bank (DSHB) (Iowa City, IA, United States) containing MHC-IIA (SC-71 dilution 1:25), MHC-IIX (6H1 dilution 1:100), and Laminin (28E dilution 1:12.5). MHC-I was left unstained to optimize the immunofluorescent signal to noise ratio and to minimize non-specific binding in the background of images. After an overnight incubation, a 3 × 5-min wash at room temperature was completed using PBS/0.1% Triton-X100 prior to and following a 1 h incubation at room temperature with the secondary antibody (2°Ab) cocktail with specific fluorophores directed to each 1°Ab. These included MHC-IIA/Alexa Fluor 350 (IgG1 dilution 1:100; Invitrogen #A21120), MHC-IIX/Alexa Fluor 488 (IgM dilution 1:500; Invitrogen #A21042), and Laminin/Alexa Fluor 555 (IgG2a dilution 1:250 #A21137). Slides were then mounted with an anti-fade mounting medium (Vectashield Hardset^TM^; Vector Laboratories, Burlingame, CA, United States) prior to microscopy imaging. Multi-channel 5 × 5 tile scanned images of entire cross-sections were captured with an incubated confocal laser scanning microscope (Zeiss LSM 880 with Airyscan; Zeiss International, Oberkochen, Germany). Images were processed for fCSA and MHC content using ImageJ software (ImageJ 1.53a, Java 1.8.0_172_64-bit, National Institutes of Health, Bethesda, MD, United States). Due to the lack of MHC-IIX fibers, only MHC-I and MHC-IIA fibers were used for the final analysis.

### Single Fiber Analysis

#### Single Fiber Isolation and Permeabilization

**FIGURE 2:**
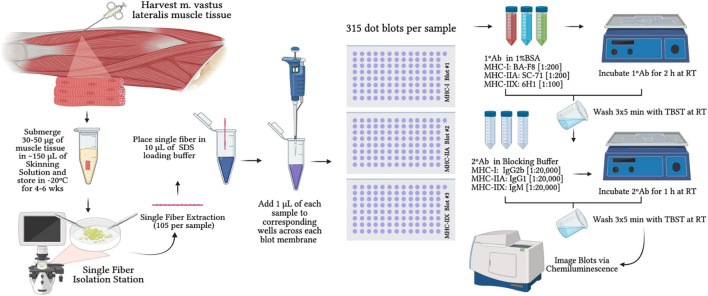
Schematic of single fiber isolation and dot blot timeline for myosin-heavy chain analysis. SDS = sodium dodecyl sulfate; MHC-I Blot #1 = immunoblot treated for myosin-heavy chain I; MHC-IIA Blot #2 = immunoblot treated for myosin-heavy chain IIA; MHC-IIX Blot #3 = immunoblot treated for myosin-heavy chain IIX; 1°Ab = primary antibody; 2°Ab = secondary antibody; 1%BSA = 1% bovine serum albumin; RT = room temperature.

Approximately 25–30 mg of tissue from each biopsy was placed in skinning solution for later analyses of permeabilized single muscle fibers to assess MHC content. Our chemical based skinning solution contained (in mM) 125 potassium propionate, 2.0 EGTA, 4.0 ATP, 1.0 MgCl_2_, and 20.0 imidazole (pH 7.0) and 50% (vol/vol) glycerol as previously described (Trappe et al., 2000b). A total of 105 ± 4 single muscle fibers that were 1–3 mm in length were isolated from tissue bundles using jeweler’s forceps. The number of single fibers used for analysis is justified by recent work from Murach et al. (2016) showing that (a) single fiber phenotyping results are the same for 25 vs. 125 fibers; and (b) false discovery rate was 0% beyond 25 fibers. Thus, any analysis >25 fibers can reliably estimate fiber type distribution of a larger sampling of fibers (Murach et al., 2016). Single fibers were placed into individually labeled 0.6-mL microcentrifuge tubes containing 10 μL of sodium dodecyl sulfate (SDS) loading buffer (0.125 M Tris–HCl, 10% glycerol, 4% SDS, 4 M urea, 10% 2-mercaptoethanol, 0.001% bromophenol blue, pH 6.8 diluted 2:1 with 1× Tris–HCl [pH 6.8]) (Christiansen et al., 2019). Samples remained in SDS loading buffer at room temperature for at least 2 h prior to immunoblotting and then placed in −20° for storage (Figure 2).

#### Single Fiber Phenotyping

An immunoblotting dot blot protocol previously described (Christiansen et al., 2019; Lamboley et al., 2020) was modified and used to determine pure MHC-I, -IIA, -IIX, and hybrid-I/IIA content of single fibers. Hybrid MHC-IIA/IIX fiber type determination was not possible with this method due to MHC-IIX antibody overstaining and non-specific binding as explained by Christiansen et al. (2019) To begin dot blotting, samples were solubilized by vortexing for ∼5 s. Three PVDF membranes cut to 8 cm × 13 cm were activated with 100% methanol and equilibrated in transfer buffer. The wet membranes were placed on top of a single piece of dry filter paper and a 96 well wafer was affixed atop each membrane (Figure 2). For each sample per subject, 1 μL of each fiber was applied to the same corresponding well of each wet membrane and allowed to dry. The dry membranes were then reactivated with 100% methanol and equilibrated in transfer buffer (25 mM Tris, 192 mM glycine, pH 8.3 and 20% methanol). Per our modifications, each blot was placed inside individual PerfectWestern^TM^ blot boxes (GenHunter Corporation, Nashville, TN, United States) to optimize each MHC signal, avoid Ab cross-activity, and to eliminate stripping of proteins. Each blot was quick washed for ∼30 s three times in Tris-buffered saline containing 0.1% Tween20 (TBST), and then placed in blocking buffer (5% non-fat dry milk in TBST) for 5 min. Following blocking, membranes were rinsed with TBST and incubated individually for 2 h with gentle rocking with 1°Ab obtained from DSHB diluted in 1% BSA/PBST. Each 1°Ab corresponded to labeled blot boxes for blot box #1 for MHC-I (BA-F8 dilution 1:200), blot box #2 for MHC-IIA (SC-71 dilution 1:200), and blot box #3 for MHC-IIX (6H1 dilution 1:100). After 1°Ab incubations, membranes were washed 3 × 5 min with TBST and then incubated in goat anti-mouse IgG horseradish peroxidase 2°Ab at room temperature while gentle rocking for 1 h. Each 2°Ab was obtained from Invitrogen and diluted in blocking buffer corresponding to blot box #1 for MHC-I (IgG2b; diluted 1:20,000), blot box #2 for MHC-IIA (IgG1; diluted 1:20,000), and blot box #3 for MHC-IIX (IgM; diluted in 1:20,000). Lastly, the 3 × 5 min wash was repeated and membranes were individually exposed to clarity enhanced chemiluminescence reagent (SuperSignal^TM^ West Dura Extended Duration Substrate; Thermo Fisher Scientific Inc.) for molecular imaging (ChemiDoc^TM^ XRS; Bio-Rad Laboratories, Inc.) using Immun-Star HRP settings in ImageLab^TM^ software (Bio-Rad Laboratories, Inc.).

**FIGURE 3:**
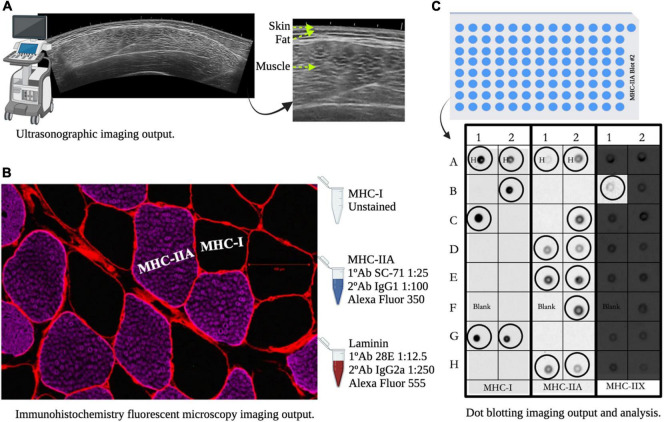
Muscle measurements for B-mode ultrasonography whole muscle imaging, immunohistochemical muscle fiber analysis, and single fiber dot blot analysis. Panel **(A)** shows image differences between skin, subcutaneous fat, and skeletal muscle mass of the vastus lateralis. Panel **(B)** shows cell border staining (Laminin), unstained myosin-heavy chain (MHC)-I fibers (Black) and fibers that positively stained for MHC-IIA (Magenta). Panel **(C)** shows dot blotting output and analysis procedures: circled dot = fiber detected, H = hybrid MHC-I/IIA detection, and Blank = example of no fiber detection.

It was possible to determine pure MHC-I, -IIA, and -IIX content per each blot probed for specific 1° Ab and 2°Ab as well as hybrid MHC-I/IIA (Figure 3). If no MHC protein was present, the corresponding well for all 3 blots produced a blank which may have indicated an unsuccessful collection of a fiber. Additionally, a process of elimination was used to determine MHC content as described previously (Christiansen et al., 2019). Christiansen et al. (2019) determined that the quantification of MHC-I, -IIA, -IIX, and -I/IIA is a reliable and valid phenotyping method compared to Western Blots using a total of 40 single fibers. In this study, we compared the fiber types of ∼3,000 fibers from immunohistochemical (IHC) cross-sections to ∼3,000 single fibers which produced a very strong relationship between the two methods (*r*^2^ = 0.94) per pure fiber content. It appeared that the pure MHC output was similar between each method; however, the dot blot protocol produced higher sensitivity for I/IIA hybrid fibers whereas IHC output showed greater sensitivity for revealing possible IIA/IIX hybrid fibers. Our methodology is in agreeance with previous literature showing that single fiber phenotyping with strength and power athletes can accurately assess the presence of pure and hybrid fibers compared to the common over- and under-estimations produced from traditional IHC analyses (Serrano et al., 2019). A recent study by Lamboley et al. (2020) also used dot blot phenotyping to confirm force output for MHC-I and MHC-II muscle fibers and suggest that the data of both methods were in 100% agreement.

### Muscle Messenger RNA and MicroRNA Analyses

Total RNA was extracted from ∼20 mg of tissue from the vastus lateralis biopsy using the AllPrep^®^ DNA/RNA/miRNA Universal Kit (QIAGEN GmbH, Hilden, Germany) following the manufacturer’s instructions. Briefly, frozen muscle samples were homogenized on ice in Lysing Matrix D tubes (MP Biomedicals, Irvine, CA, United States) using the Cool Prep 24 homogenizer (MP Biomedicals) followed by RNA purification. RNA quality was verified spectrophotometrically using the A_260_/A_280_ ratio ≥1.8 determined using a Nanodrop 2000 (Thermo Scientific, Rockford, IL, United States). Reverse transcription of RNA-to-complimentary DNA (cDNA) was conducted using a High-Capacity reverse transcription kit (Life Technologies, Carlsbad, CA, United States) performed with a GeneAmp^®^ PCR System 9700 (Applied Biosystems; Foster City, CA, United States). cDNA was quantified using a nanodrop and aliquots were stored at −20°C for real time-polymerase chain reaction (RT-PCR) analysis. Genes and miRNAs of interest were based on previous literature demonstrating significant roles in hypertrophy, strength, and fiber type changes relative to a given training stimulus or controls (D’Souza et al., 2017, 2019; Bjørnsen et al., 2019a). Genes selected for analysis were the following: PAX7, MSTN, MyoD, MyoG, SOX6, MYH7, MYH2, and MYH1 (Supplementary Table 1). Additionally, endoplasmic reticulum membrane protein complex subunit 7 (EMC7), charged multivesicular body protein 2A (CHMP2A), and chromosome 1 open reading frame 43 (C1orf43) were used as housekeeping genes for reference (Eisenberg and Levanon, 2013; D’Souza et al., 2017). Target miRNAs selected for quantitation were miR-23a-5p, -133a-3p, -206, -451a, -486-5p, -499a-3p, while miR-186-5p and -361-5p were used for reference (TaqMan^®^ Advanced miRNA Assays, Thermo Fisher Scientific) (Vandesompele et al., 2002; McCarthy, 2011; D’Souza et al., 2017). All mRNA primer sequences were designed using a BLAST software and determined based on previously published primer reports (Supplementary Table 2) (D’Souza et al., 2017).

Quantitative RT-PCR (qPCR) analysis of mRNA for target genes was conducted using Power SYBR^TM^ Green I Master Mix (Applied Biosystems) using gene specific primers. Additionally, target miRNAs analyses were conducted using TaqMan^®^ Fast Advanced Master Mix (Applied Biosystems). Samples and reagents were loaded in a MicroAmp Fast-Optical 96 Well Reaction Plate and run in triplicate. Plates were analyzed on a 7500 Fast RT-PCR System (Applied Biosystems). Relative mRNA levels were normalized to C_*T*_ calculations from housekeeping genes. Standard and melting curves were performed for every target to confirm primer efficiency and single-product amplification. The abundance of mRNA was measured using the 2^–ΔΔ*CT*^ method (Livak and Schmittgen, 2001; Schmittgen and Livak, 2008).

### Statistical Analyses

The results are presented as mean values and standard deviations (mean ± SD). A 2 × 2 repeated measures analysis of variance (ANOVA; Group × Time) was used to test all performance and physiological variables. Significant main effects were followed by *post hoc* pairwise comparisons using a Holm-Bonferroni adjustment. Effect sizes were determined using Hedge’s g (*g*) and classified as small ≤0.20, medium between >0.20 and <0.60, and large >0.60 (Hedges, 1980). Alpha level for significance was defined as *p* ≤ 0.05. All analyses were performed using IBM SPSS Statistics v.27 (SPSS Inc., Chicago, IL, United States) and Microsoft Excel v16.49 (Microsoft Corporation, Redmond, WA, United States).

## Results

### Training Volume-Load, Monotony, and Strain

**FIGURE 4:**
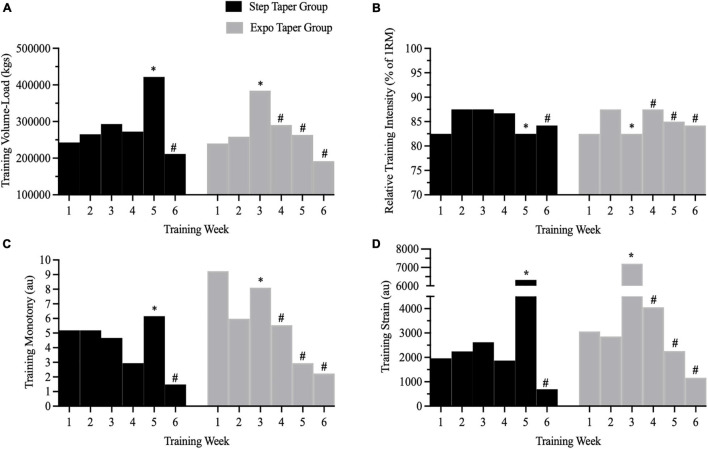
Training metrics from all work completed across the 6-week peaking program. Panels represent **(A)** training volume-load, **(B)** relative training intensity, **(C)** training monotony, and **(D)** training strain. ^∗^ = planned overreach week; # = taper weeks.

There was a significant group by time interaction (*p* < 0.001), and main time effect (*p* < 0.001) for training volume-load. *Post hoc* analyses revealed volume-load during weeks 2–5 was significantly greater than week 1 volume load within each group (*p* < 0.05). However, week 6 volume-load was significantly less than week 1 volume-load in the step (*p* < 0.001) and exponential taper group (*p* < 0.001). Further, volume-load was significantly different between groups during week 3 (*p* = 0.019) and week 5 (*p* = 0.001) corresponding to the overreach week during the exponential and step taper, respectively. However, total volume-load completed during the 6-week program (step taper: 213,323 ± 50,066 kg vs. exponential taper: 203,568 ± 35,260 kg) was not significantly different between groups (Figures 4A,B).

There was a significant group by time interaction (*p* = 0.016), and main time effect (*p* < 0.001) for training monotony. *Post hoc* analyses revealed training monotony was significantly lower during weeks 2 (*p* = 0.023), 4 (*p* = 0.021), 5 (*p* = 0.025), and 6 (*p* < 0.001) compared to week 1 in the exponential taper group. However, only training monotony during week 6 (*p* = 0.023) was lower than week 1 in the step taper group. Further, training monotony was significantly greater during week 5 in the step taper group compared to the exponential taper group (*p* = 0.027). Total training monotony during the 6-week program was not significantly different between groups (Figure 4C).

There was a significant group by time interaction (*p* < 0.001), and main time effect (*p* < 0.001) for training strain. *Post hoc* analyses revealed training strain was significantly greater during week 3 (*p* = 0.002), and lower during week 6 (*p* = 0.006) compared to week 1 in the exponential taper group. Training strain was significantly greater during week 5 (*p* = 0.007), and lower during week 6 (*p* = 0.05) compared to week 1 in the step taper group. Further, training strain was significantly greater during week 3 (*p* = 0.009) in the exponential taper group compared to the step taper group, but vice-versa during week 5 (*p* = 0.028) corresponding to the overreach weeks in each group. Total training strain during the 6-week program was not significantly different between groups (Figure 4D). There were no other significant main effects for training volume-load, monotony, and strain.

### Performance Assessments

**FIGURE 5:**
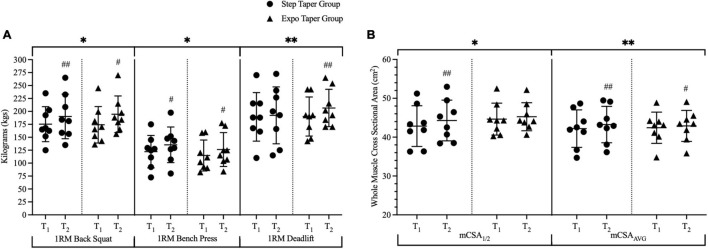
Changes over time for 1-repetition-maximum performances and whole muscle cross-sectional area. Panel **(A)** represents all lifts that were completed during 1RM testing and panel **(B)** represents the significant changes in whole muscle measurements via ultrasound. Data is represented by means ± standard deviations. Significant main time effects: ^∗^*p* ≤ 0.05, ^∗∗^*p* ≤ 0.001. Significant group change from baseline: #*p* ≤ 0.05, ##*p* ≤ 0.001. T_1_ = pre-training; T_2_ = post-taper; 1RM = 1-repetition-maximum; mCSA = muscle cross-sectional area; mCSA_1/2_ = medial vastus lateralis measurement; mCSA_*AVG*_ = average of three sites for vastus lateralis measurements.

For maximal strength, there were significant main time effects for back squat 1RM (*p* < 0.001), bench press 1RM (*p* < 0.001), deadlift 1RM (*p* = 0.024), powerlifting total (*p* < 0.001), and Wilks Score (*p* < 0.001) (Figure 5A and Table 3). *Post hoc* pairwise comparisons revealed statistically significant increases in back squat 1RM (*p* = 0.002, *g* = 0.37; *p* < 0.001, *g* = 0.54), bench press 1RM (*p* < 0.001, *g* = 0.38; *p* < 0.001, *g* = 0.35), powerlifting total (*p* = 0.003, *g* = 0.25; *p* < 0.001, *g* = 0.48), and Wilks Score (*p* = 0.003, *g* = 0.36; *p* < 0.001, *g* = 0.55) following the step-taper and the exponential taper, respectively. However, deadlift 1RM (*p* = 0.009, *g* = 0.48) significantly increased following the exponential taper only. There was as a significant main time effect for SJ PPa (*p* = 0.001), but not for SJH or ISQ IPFa (Table 3). *Post hoc* pairwise comparisons revealed statistically significant increases for SJ PPa (*p* = 0.001, *g* = 0.84) following the exponential taper only. No significant interactions or main effects were observed for any other performance measure.

**TABLE 3:** Performance, body composition, and muscle morphometry.

	Pre-training (T_1_)			Post-taper (T_2_)		
	Combined groups	Step-taper group	Exponential-taper group	Combined groups	Step-taper group	Exponential-taper group
**Performance**						
1RM back squat (kg)	174.7 ± 33.4	175.3 ± 33.8	174.1 ± 35.3	192.3 ± 38.2**	190.2 ± 43.1*	194.5 ± 35.5**
1RM bench press (kg)	118.5 ± 29.9	122.2 ± 31.3	114.8 ± 29.9	130.8 ± 32.8**	135.4 ± 34.5**	126.3 ± 32.8**
1RM deadlift (kg)	189.8 ± 41.2	189.4 ± 47.0	190.1 ± 37.8	199.5 ± 45.6*	192.3 ± 55.0	206.6 ± 36.1*
Total (kg)	483.0 ± 97.9	486.9 ± 108.0	479.0 ± 94.1	522.6 ± 109.5**	517.9 ± 127.5*	527.3 ± 96.8**
Wilks Score (au)	328.3 ± 46.8	328.9 ± 44.2	327.7 ± 52.4	353.4 ± 53.7**	347.8 ± 55.7^∗^	359.1 ± 54.7**
IPFa (N)	125.6 ± 14.4	122.7 ± 14.3	128.5 ± 14.8	128.2 ± 15.7	125.3 ± 18.5	131.0 ± 13.1
SJH (cm)	31.0 ± 8.8	30.9 ± 11.2	31.1 ± 6.4	32.1 ± 7.6	31.8 ± 10.1	32.4 ± 4.5
SJPPa (W)	220.1 ± 38.3	222.8 ± 49.9	217.4 ± 25.3	234.8 ± 33.7	231.8 ± 44.4	237.9 ± 20.8
**Body composition**						
Body mass (kg)	89.8 ± 21.4	88.6 ± 19.0	91.1 ± 24.9	91.4 ± 22.3*	90.2 ± 20.0*	92.5 ± 25.7*
Fat mass (kg)	23.2 ± 13.3	21.9 ± 11.7	24.4 ± 15.4	24.0 ± 12.9	22.9 ± 11.4	25.1 ± 14.9
Skeletal muscle mass (kg)	33.8 ± 6.4	33.8 ± 5.9	33.9 ± 7.2	34.2 ± 6.5	34.0 ± 6.1	34.4 ± 7.3
Fat mass index (kg/m^2^)	7.6 ± 4.4	7.2 ± 3.8	8.0 ± 5.1	7.9 ± 4.2	7.5 ± 3.7	8.2 ± 5.0
Fat free mass index (kg/m^2^)	21.8 ± 2.6	21.9 ± 2.6	21.7 ± 2.8	22.1 ± 2.8	22.1 ± 2.8	22.0 ± 7.2
Total body water (I)	49.1 ± 8.4	49.1 ± 7.7	49.1 ± 9.5	49.6 ± 8.8	49.6 ± 8.0	49.6 ± 10.0
Extracellular water (I)	19.3 ± 3.3	19.3 ± 3.0	19.3 ± 3.8	19.4 ± 3.5	19.5 ± 3.1	19.4 ± 4.2
**Muscle morphometry**						
mCSA_*proximal*_ (cm^2^)	45.7 ± 5.1	46.2 ± 5.2	45.3 ± 5.2	46.4 ± 4.6	47.0 ± 5.0	45.7 ± 4.3
mCSA_*medial*_ (cm^2^)	43.7 ± 4.6	42.9 ± 5.2	44.6 ± 4.1	44.8 ± 4.4	44.3 ± 5.2	45.3 ± 3.6
mCSA_*distal*_ (cm^2^)	37.5 ± 5.2	37.6 ± 5.2	37.3 ± 5.6	38.1 ± 5.3	38.4 ± 4.8	37.7 ± 6.1
fCSA MHC-I (μm^2^)	6831.6 ± 730.2	6557.7 ± 802.5	7066.4 ± 624.9	7160.7 ± 682.6	6764.9 ± 788.9	7500.0 ± 349.7
fCSA MHC-IIA (μm^2^)	7461.3 ± 728.4	7268.5 ± 974.5	7626.8 ± 447.9	7890.9 ± 560.9	8169.7 ± 575.9	7651.9 ± 456.6

*p < 0.05; **p < 0.001 denote significant difference from pre- (T_1_) to post-training (T_2_).

*1RM = 1-repetition-maximum; IPFa = isometric peak force allometrically scaled to body mass; SJH = squat jump height; SJPPa = squat jump peak power allometrically scaled to body; mCSA = whole muscle cross-sectional area; fCSA = muscle fiber cross-sectional area proximal = cross-sectional area at proximal vastus lateralis; medial = cross-sectional area middle vastus lateralis; distal = cross-sectional area distal vastus lateralis; MHC-I = myosin-heavy chain I isoform; MHC-IIA = myosin-heavy chain IIA isoform.*

### Body Composition Assessments

For body composition assessments, there were significant main time effects for body mass (*p* = 0.005), FM (*p* = 0.002), and FMI (*p* = 0.002) (Table 3). *Post hoc* pairwise comparisons revealed statistically significant increases in the step taper group for body mass (*p* = 0.021, *g* = 0.08), FM (*p* = 0.005, *g* = 0.08), and FMI (*p* = 0.010, *g* = 0.08), but only significant increases in the exponential taper group for body mass (*p* = 0.047, *g* = 0.05) and FMI (*p* = 0.038, *g* = 0.05). No significant interactions or main effects were observed for any other body composition measure.

### Skeletal Muscle Assessments

At the whole muscle level, there were significant main time effects for mCSA_1/2_ (*p* = 0.006) and mCSA_*avg*_ (*p* < 0.001) (Figure 5B). *Post hoc* pairwise comparisons revealed significant increases in mCSA_1/2_ (*p* = 0.007, *g* = 0.26) and mCSA_*avg*_ (*p* < 0.001, *g* = 0.21) following the step taper, and significant increases in mCSA_*avg*_ (*p* = 0.047, *g* = 0.11) following the exponential taper. Main time effects for mCSA_1__/__3_ (*p* = 0.077, *g* = 0.13) and mCSA_2__/__3_ (*p* = 0.067, *g* = 0.11) exhibited small effect sizes, but did not reach significance. There were no significant interactions or main effects observed for other whole muscle measurements.

**FIGURE 6:**
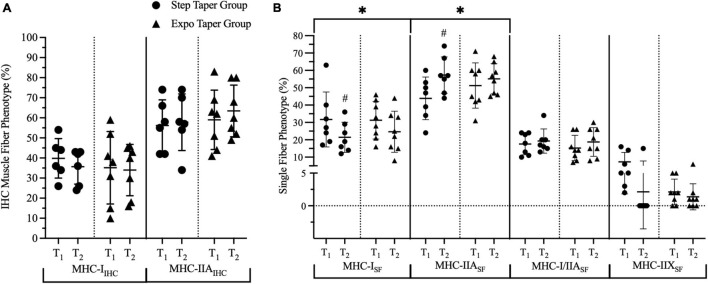
Phenotype composition based on myosin-heavy chain content. Panels represent fiber composition per **(A)** immunohistochemistry for type I and IIA fibers and **(B)** single fiber analyses for type I, IIA, I/IIA, and IIX. Data is represented by means ± standard deviations. Significant main time effects: ^∗^*p* ≤ 0.05. Significant group change from baseline: #*p* ≤ 0.05. T_1_ = pre-training; T_2_ = post-taper; IHC = immunohistochemistry; SF = single fiber; MHC = myosin-heavy chain; MHC-I = myosin-heavy chain I; MHC-IIA = myosin-heavy chain IIA; MHC-I/IIA = myosin-heavy chain I/IIA hybrid; MHC-IIX = myosin-heavy chain IIX.

At the muscle fiber level using IHC analysis, there was a significant group by time interaction for MHC-IIA fCSA (*p* = 0.014) (Figure 6A). There were also significant main time effects for fCSA_*avg*_ (*p* = 0.020) and MHC-IIA fCSA (*p* = 0.010). *Post hoc* pairwise comparisons revealed statistically significant increases in fCSA_*avg*_ (*p* = 0.010, *g* = 0.90) and MHC-IIA fCSA (*p* = 0.002, *g* = 1.07) only following the step taper.

At the isolated single muscle fiber level using immunoblot dot blotting analysis, there were significant main time effects for MHC-I_*SF*_% (*p* = 0.015) and MHC-IIA_*SF*_% (*p* = 0.033) (Figure 6B). *Post hoc* pairwise comparisons revealed a significant decrease in MHCI_*SF*_% (*p* = 0.037, *g* = 0.78), and a significant increase in MHC-IIA_*SF*_% (*p* = 0.023, *g* = 1.11) only following the step taper. The main time effect for MHC-IIX_*SF*_% (*p* = 0.087, *g* = 0.63) exhibited a large effect size, but did not reach statistical significance.

**FIGURE 7:**
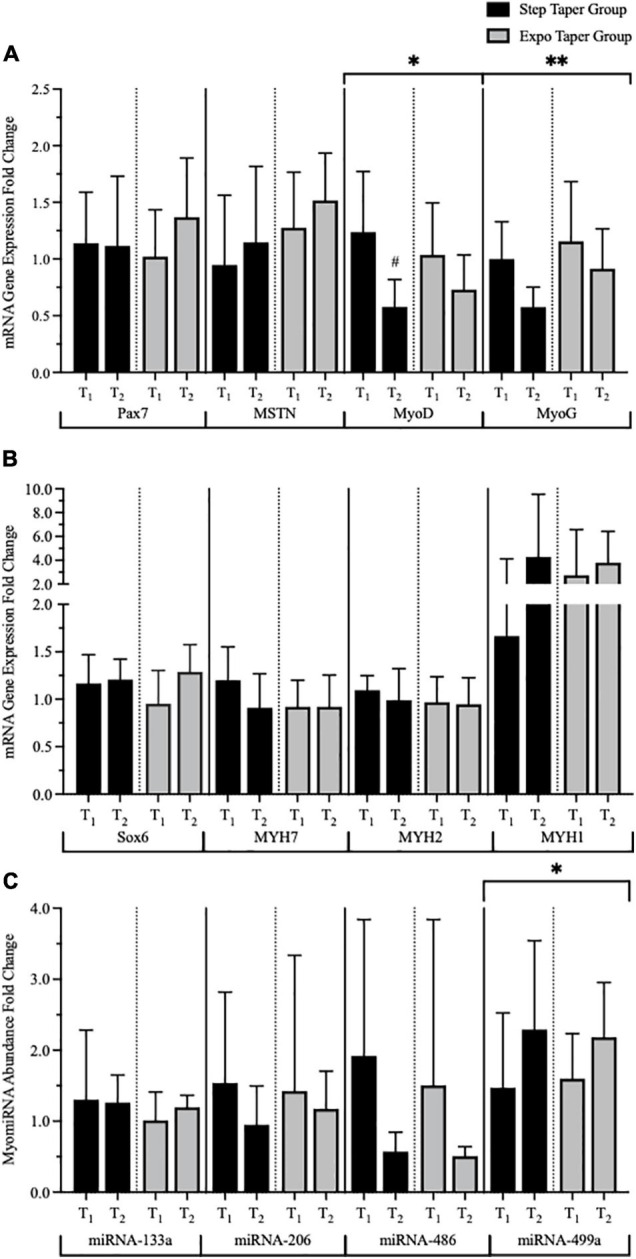
Messenger RNA (mRNA) gene expression and myomiRNA abundance over time. Panels represent **(A)** myogenic mRNA gene expression, **(B)** phenotype mRNA gene expression, and **(C)** myomiR abundance. Data is represented by means ± standard deviations. Significant main time effects: ^∗^*p* ≤ 0.05, ^∗∗^*p* ≤ 0.001. Significant group change from baseline: #*p* ≤ 0.05. T_1_ = pre-training; T_2_ = post-taper; Pax7 = Paired Box 7; MSTN = Myostatin; MyoD = Myogenic Differentiation 1; MyoG = Myogenin; Sox6 = SRY-Box Transcription Factor 6; MYH7 = β-myosin-heavy chain for slow twitch skeletal muscle; MYH2 = myosin-heavy chain 2; MYH1 = striated muscle myosin-heavy chain 1. miRNA = microRNA.

At the molecular level using mRNA and miRNA analyses, there were significant main time effects for MyoD (*p* = 0.002), MyoG (*p* = 0.037), and miR-499a (*p* = 0.033) (Figure 7). *Post hoc* pairwise comparisons revealed significant decreases in MyoD (*p* = 0.002, *g* = 1.60) only following the step taper. Main time effects for Sox6 (*p* = 0.053, *g* = 0.65), MYH1 (*p* = 0.08, *g* = 0.49), MSTN (*p* = 0.053, *g* = 0.39), and miR-486 (*p* = 0.06, *g* = 0.99) exhibited moderate to large effect sizes, but did not reach statistical significance. No additional significant myocellular changes were observed.

## Discussion

This is the first study to compare performance changes coupled with skeletal muscle adaptations at the whole muscle, muscle fiber, and molecular levels between two tapering models in powerlifters. Our main findings indicate equated volume-loads over 6-weeks produced similar outcomes following the step and exponential tapers with some exceptions. Changes in 1RM performance were similar between taper models; however, changes in deadlift 1RM favored the exponential taper. Secondary performance assessments (i.e., SJH and ISQ IPF) did not appear to be as sensitive to the peaking program compared to 1RMs. Skeletal muscle can be positively augmented based on mCSA increases following both tapers, but single fiber MHC phenotype changes appear to favor the step taper. However, the overall lack of differences between taper models is likely attributed to the similar total workload completed by both groups, whereas the distribution of work may explain the favorable muscular adaptations observed in the step taper group. Thus, the findings from these two taper models warrant further discussion.

Tapering prior to competition has been shown to improve competition and laboratory-based performances (Mujika et al., 2002; Mujika and Padilla, 2003; Seppänen and Häkkinen, 2020; Travis et al., 2020b,c) along with enhancing physiological factors (Trappe et al., 2000a; Neary et al., 2003; Coutts et al., 2007; Murach et al., 2014; Zaras et al., 2014; Bazyler et al., 2018; Travis et al., 2020b). A systematic review by Travis et al. (2020c) and an experimental study by Seppänen and Häkkinen (2020) provide evidence to suggest a ∼50% volume-load reduction may be ideal to enhance or maintain maximal strength. However, volume-load reductions typically follow a normal training period or a planned overreach microcycle, both of which may influence the magnitude of performance improvement. To taper effectively, the current evidence supports using a planned overreach period prior to tapering. However, this assumes a sufficient stimulus and recovery period is provided to elicit a super-compensation effect (Aubry et al., 2014; Seppänen and Häkkinen, 2020; Travis et al., 2020b; Williams et al., 2020). Training programs implementing a 100–200% increase in volume-load followed by a taper have been shown to improve competition and laboratory based performances (i.e., bench press, snatch, clean-and-jerk, and jump height), and biochemical markers of training stress (i.e., cortisol, creatine kinase) in weightlifters, powerlifters, and track and field throwers (Warren et al., 1992; Fry et al., 1993; Stone and Fry, 1998; Williams et al., 2020). Similarly, the current results indicate 1RM strength improvements can be achieved following a 1-week planned overreach with a 3-week exponential taper or a 1-week planned overreach with a 1-week step taper in strength athletes.

While 1RMs for back squat (step: 8%Δ vs. expo: 10%Δ) and bench press (step: 10%Δ vs. expo: 9%Δ) improved similarly in both groups, 1RM deadlift performance favored the 3-week exponential taper (8%Δ) compared to the step taper group (1%Δ). Prior exposures to intensified training, such as overreaching microcycles, seem to reduce the likelihood of performance decrements in experienced athletes possibly due to a repeated bout effect (Stone and Fry, 1998; Pistilli et al., 2008). Nonetheless, it is possible the 1-week step taper did not provide sufficient recovery time for some athletes following the planned overreach week, particularly for deadlift. Interestingly, back squat, bench press, and deadlift exhibit similar recovery patterns in strength-trained males following four sets of each lift performed to failure using 80% 1RM (Belcher et al., 2019), and eight sets of two repetitions of back squat and deadlift performed at 95%1RM (Barnes et al., 2019). Nonetheless, these studies did not examine the cumulative effects of repeated training sessions on deadlift performance. Further, anecdotal reports from powerlifters (Pritchard et al., 2016; Grgic and Mikulic, 2017) and strongman competitors (Winwood et al., 2018) claim the deadlift requires a longer recovery period between final training and competition compared to the other lifts. Thus, a 3-week exponential taper may be warranted following a planned overreach to facilitate recovery-adaptation and enhance performance concurrently across each power lift.

Furthermore, SJ PPa (step: 4%Δ vs. expo: 9%Δ) was the only laboratory-based performance measure that increased following the 6-week peaking phase, specifically following the exponential taper. Although peak power calculations are heavily influenced by body mass (Cormie et al., 2007), when allometrically scaled for body mass, SJ PPa still improved following the exponential taper. This finding likely reflects an improved ability to generate high ground reaction forces relative to the athlete’s body mass. This is reflected by strong relationships observed between 1RM back squat scaled for body mass and jumping performance (Jacobson, 2015). Thus, it is likely that improvements in lower body maximal strength contributed to the increased SJ PPa following the exponential taper. Interestingly, improvements in SJH and SJ PPa have been repeatedly observed in weightlifters following an overreach and 3-week exponential taper (Bazyler et al., 2018; Travis et al., 2018, 2020b). Nonetheless, this study did not observe increases in SJH following either taper model. This discrepancy may be attributed to the different training programs employed by powerlifters compared to weightlifters, particularly in the current study and prior studies with weightlifters from our laboratory. Notably, weightlifters commonly train with movements (e.g., snatch, clean and jerk, clean pulls, and mid-thigh pulls) emphasizing “triple extension” of the hips, knees, and ankle joints, which exhibit a high degree of task specificity to jumping. Additionally, it is also possible the 3-week exponential taper provided greater recovery time to enhance jumping ability following the overreach compared to the 1-week step taper. Nonetheless, it appears the “transfer of training effect” from the powerlifting-oriented training in the current study to SJ performance is smaller than that observed previously in weightlifters during a taper (Bazyler et al., 2018; Travis et al., 2020b).

Despite improvements in 1RM squat, there were no improvements in ISQ IPF following either taper. A strong relationship (*r* = 0.84) has been observed between 1RM back squat and ISQ IPF at a 90° knee angle (Bazyler et al., 2015). ISQ IPF has also been shown to improve concurrently with 1RM back squat following 7 weeks of back squat training in strength-trained males (Bazyler et al., 2014). These discrepancies may be explained by the higher initial relative strength levels of participants in the current study (squat to body mass ratio: 1.94 ± 0.34) compared to the aforementioned study (1.73 ± 0.19). It is well established that changes in maximal strength following strength training are inversely related to initial relative strength levels (Ahtiainen et al., 2003; Ishida et al., 2020, 2021). Thus, given the higher degree of task specificity of back squat training to 1RM back squat compared to isometric squats, it is possible 1RMs provide a more sensitive measure to assess changes in maximal strength, particularly for subjects with greater initial relative strength. Nonetheless, differences in training program design, selection and order of testing procedures could also explain the differences in outcomes for ISQ IPF between studies.

There is a direct relationship between increases in mCSA and the ability to produce force (Häkkinen et al., 1991; Gabriel et al., 2006; Schoenfeld et al., 2014; Enoka and Duchateau, 2017; Seppänen and Häkkinen, 2020). Muscular adaptations are highly influenced by the prescription of training variables such as volume-load and training intensity (Schoenfeld et al., 2014; Bjørnsen et al., 2019a; Haun et al., 2019; Travis et al., 2020a). In the current study, it appears that training volume-load prescription was sufficient to elicit hypertrophic adaptations at the whole muscle level in conjunction with 1RM strength improvements. Schoenfeld et al. (2014) demonstrated similar improvements in biceps brachii muscle thickness in strength-trained males following equated volumes of bodybuilding-styled and powerlifting-styled training over 8 weeks; however, improvements in maximal strength favored the powerlifting-styled training. Similarly, meta-analytic results from Schoenfeld et al. (2017) demonstrate improvements in maximal strength are best achieved with high loads (>60% 1RM) compared to low loads (≤60% 1RM) while muscle hypertrophy can be achieved across a broad spectrum of loads. Thus, our results for 1RM and whole muscle size changes are consistent with previous studies when volume-loads are equated between training programs, and heavy loads are used in training. However, studies with weightlifters, throwers, and strength-trained individuals have reported no changes or small decreases in vastus lateralis mCSA following a taper (Häkkinen et al., 1991; Zaras et al., 2014, 2016; Bazyler et al., 2017, 2018; Suarez et al., 2019; Seppänen and Häkkinen, 2020; Travis et al., 2020b). Nonetheless, these studies have typically implemented either: (a) smaller increases in volume-load during the overreach (<150%) or (b) normal training followed by a 1–4 week taper consisting of larger reductions (≥50%) in volume-load. Despite matched reductions in volume-load (−50%) during the final week of the study, in the current study, mCSA increases were relatively larger across all measurement sites (proximal: 1.8% vs. 1.0%, middle: 3.2% vs. 1.4%, distal: 2.2% vs. 0.95%, and average: 2.4% vs. 1.1%) in the step taper compared to the exponential taper group. Changes in fCSA_*avg*_ (8.6% vs. 1.7%) and MHC-IIA fCSA (11.0% vs. 0.33%) were even more pronounced favoring the step taper group. These results may be due to the timing of the overreach relative to post-training testing. Specifically, the overreach may have provided a greater hypertrophic stimulus closer to post-training testing in the step-taper (2 weeks prior) compared to the exponential taper (4 weeks prior). Indeed, previous studies observing decreases in vastus lateralis mCSA have attributed the decreases in muscle size to the prolonged reduction in volume-load during 3-week exponential tapers. Nonetheless, there was still a small, significant increase in mCSA_*avg*_ following the exponential taper, which may partly be due to the larger overreach (+150%) implemented in the current study compared to previous studies (+20 to 40%) (Thomas and Busso, 2005; Le Meur et al., 2013; Aubry et al., 2014; Bazyler et al., 2018; Travis et al., 2020b). Thus, these results suggest that a 1-week planned overreach followed by a 3-week exponential taper or a 1-week step taper produces significant improvements in strength athletes’ vastus lateralis whole muscle size provided the overreaching stimulus is sufficient leading into the taper.

It is well established that resistance training produces increases in skeletal muscle fCSA (Haun et al., 2019). Haun et al. (2019) recently investigated the mechanisms associated with fCSA hypertrophy after 6 weeks of high-volume training. In brief, myosin and actin content decreased despite enhanced fCSA, yet an accretion of sarcoplasmic proteins appeared to explain the observed changes. The authors purported that observed hypertrophy was attributed to sarcoplasmic hypertrophic adaptations as a result of high training volume. In our study, it is unlikely the observed fCSA hypertrophy was due to sarcoplasmic changes considering the training protocols aimed to reduce volume-load. Decades ago, Stone et al. (1983) suggested that it is possible to produce sarcoplasmic expansion and metabolic conditioning via higher-volume/lower-load training preceding lower-volume/higher-load training that will, in turn, produce more favorable strength outcomes. In agreement, Haun et al. (2019) suggest training with higher loads can proportionally increase myofibrillar protein levels and fCSA, which would, in turn, enhance ultrastructural hypertrophy leading to increases in maximal strength. Although fCSA constituents were not measured in the current study, it is plausible that increases in myofibrillar protein levels could have contributed to the observed increases in fCSA. Future investigations should examine the constituents of muscle fiber size changes following a taper in strength athletes.

Skeletal muscle is a highly plastic tissue that shows a remarkable ability to adapt to imposed demands (Schoenfeld et al., 2014), particularly at the muscle fiber level. Previous work from 1976 to 2005 (Prince et al., 1976; MacDougall et al., 1982; Tesch et al., 1984; Kadi et al., 1999; Fry et al., 2003; Eriksson et al., 2005) and two recent studies (Bjørnsen et al., 2019a; Machek et al., 2020) have characterized the fiber types of powerlifters. Powerlifters typically demonstrate the highest expression of MHC-IIA isoforms followed by MHC-I isoforms to a lesser degree, and depending on training status, potentially little to no MHC-IIX isoforms. At baseline, our data from biopsy tissue IHC analyses and isolated single fiber analyses agree with the current literature (Prince et al., 1976; MacDougall et al., 1982; Tesch et al., 1984; Kadi et al., 1999; Fry et al., 2003; Eriksson et al., 2005; Bjørnsen et al., 2019a; Machek et al., 2020). However, the isolated single fiber analyses appeared to produce a higher sensitivity yield for accurate quantitation of pure phenotype expression along with accurately identifying hybrid MHC-I/IIA expression. A recent study by Serrano et al. (2019) using both techniques, characterized the muscle fiber types of elite weightlifters and demonstrated that the single fiber method confidently identified the hybrid isoforms, whereas homogenate analyses did not. Thus, to accurately quantify fiber type at baseline and changes across both tapering and peaking protocols, the isolated single muscle fiber analysis was used in our study. Our single fiber dot blotting technique objectively identified MHC shifts toward MHC-IIA (i.e., MHC-I → MHC-I/IIA → MHC-IIA ← MHC-IIA/IIX ← MHC-IIX) from T_1_ to T_2_. The MHC shift observed in this study reflects a fiber type transition taking place in as little as 6 weeks, moving toward a preferential fiber type as a result of an effective peaking program. This is a novel finding considering no other published work has demonstrated this phenomenon with powerlifters peaking for competition. Acute training activates a distinct MHC-IIA transcriptome that results in a training-induced increase in MHC-IIA fCSA at the single fiber level (Murach et al., 2014). Murach et al. (2014) indicate an increase in MHC-IIA single fiber fCSA can augment the capacity of MHC-IIA fibers to quickly grow and improve contractile function in the lateral gastrocnemius of distance runners during a taper. Thus, the observed increases in MHC-IIA fCSA, at the whole muscle fiber level, and MHC-IIA_*SF*_% may explain the performance improvements observed, particularly following the step taper.

At the myocellular level, mRNA up- and down-regulations drive gene expression and miR abundance that can produce observable MHC isoform adaptations and growth beginning with single fibers. One of the most important gene-miR interactions is the up-regulation of SOX6 (which approached significance in the current study; *p* = 0.053), and the significant down-regulation of miR-499a. The post-transcriptional mechanisms between the SOX6 and miR-499a interaction are directly related to fiber type regulation, which have been confirmed by McCarthy (2011) and Bjørnsen et al. (2019a) The interaction observed between SOX6 and miR-499a as a result of the training stimulus provided in this study also confirms our single fiber MHC fiber typing quantitation (i.e., post-taper shift toward MHC-IIA_*SF*_). Furthermore, the transcriptional repressor SOX6 and miR-499a have been shown to regulate muscle mass, and in part directly influence MSTN. Interestingly, our results showed an up-regulation of MSTN, which approached significance (*p* = 0.053) while other myogenic factors MyoD and MyoG were significantly down-regulated. It is important to note that increased muscle mass can occur regardless of MSTN expression levels (Bjørnsen et al., 2019b). Paradoxically, MSTN mRNA expression is greater in larger muscle fibers (Carlson et al., 1999). Thus, increases in MSTN expression may correspond to the fCSA increases observed in the present study. Additionally, myogenic markers have been shown to play a significant role in MHC composition (Mozdziak et al., 1998). In mature muscle, MyoD and MyoG typically possess low expression levels (Mozdziak et al., 1998). However, acute bouts of resistance training can significantly increase expression of MyoD and MyoG mRNA corresponding to increases in MHC-I, -IIA, and -IIX mRNA expression (Willoughby and Nelson, 2002). Nonetheless, decreases in resting MyoD and MyoG expression following the taper may reflect a molecular adaptation of a muscle that already achieved full recovery prior to the T_2_ biopsy. Despite the limited molecular changes following the taper, our data aligns with other reports on gene expression and miRNA abundance changes in resting conditions in powerlifters (D’Souza et al., 2017; Bjørnsen et al., 2019a). However, these results should be interpreted with caution considering molecular measurements are transient, and these data may not fully reflect the myocellular response immediately post-taper. Recent findings by Vann et al. (2021) demonstrate the transient nature of molecular assessments even with biopsy measurements taking place 24 h post-intervention.

There are a few limitations that should be considered when interpreting the results of this study. First, it is unknown whether edema contributed to mCSA changes measured via ultrasonography following the taper. Nevertheless, ultrasound images were collected at least 72 h following 1RM testing at both testing time points. While we could have implemented additional measurements such as echo intensity in an attempt to identify muscle swelling, the validity of such measurement is questionable (Yitzchaki et al., 2019). Second, we did not account for sarcoplasmic myofibrillar protein content, which could have differentially influenced the fCSA measurements. Additionally, we did not control for total caloric or macronutrient intake (e.g., protein and carbohydrate consumption) throughout the study, which could have influenced our molecular muscle measurements, particularly at the gene and miR levels (Roy and Tarnopolsky, 1998; Machek et al., 2020). Nonetheless, dietary intake was standardized in the 48 h prior to both muscle biopsy time points. Also, it was not possible to standardize subjects’ training prior to the 6-week peaking phase; however, all subjects consistently trained for powerlifting over the year leading up to the study. Lastly, it is important to consider the muscle tissue analyses only reflected a specific snapshot in time from when the tissue was extracted. Specifically, after the taper, athletes rested for 2 days before 1RM assessments followed by 3 days of rest before muscle biopsies. Therefore, it is possible that our muscle tissue results are more reflective of a “tapered post-competition” rested state. Nevertheless, future investigators may repeat our study design and replace or precede 1RM assessments with a muscle biopsy to assess the skeletal muscle environment in a peaked state.

## Conclusion

Overall, this study provides novel evidence toward an enhanced neuromuscular profile following tapering in strength athletes. Increases in powerlifting performance following the step and exponential tapers appeared to be mediated by whole muscle, single muscle fiber, and myocellular adaptations. Specifically, increases in mCSA, fCSA, and MHC-IIA fCSA favored the step taper. Increases in MHC-IIA content with concomitant decreases in MHC-I and -IIX content were also observed following the step taper. These myosin isoform shifts toward the MHC-IIA phenotype appear to be related to changes in underlying myocellular signaling (i.e., Sox6 up-regulation and miRNA-499a down-regulation) responsible for fiber-type transitions. Thus, planning an overreach close to competition, followed by a short, step taper may support a more favorable environment to induce fast-twitch fiber adaptations compared to an overreach planned further from competition followed by an exponential taper. Nonetheless, it is possible that the 1-week step taper did not provide sufficient recovery time for some athletes following the overreach, particularly for deadlift and squat jump performance. This study also provides direct evidence for short-term skeletal muscle plasticity at all measurable levels, and subsequent potentiating effects on maximal strength performance following a taper in strength athletes. Based on these findings, we recommended strength athletes use a 1-week overreach where volume-load is increased by ≥150% followed by a step or an exponential taper where training volume-load is reduced by ∼50% over a duration of 1–3 weeks to promote a myocellular environment favorable to fast-twitch skeletal muscle adaptations and to enhance maximal strength.

## Data Availability Statement

The original contributions presented in the study are included in the article/Supplementary Material, further inquiries can be directed to the corresponding author/s.

## Ethics Statement

This study involving human participants was reviewed and approved by East Tennessee State University Institutional Review Board. The participants provided their written informed consent to participate in this study.

## Author Contributions

SKT and KZ performed the experiments and designed all primers. SKT, KZ, and CB analyzed the data. SKT, IM, and CB drafted the manuscript. All authors critically evaluated and contributed to the manuscript.

## Conflict of Interest

The authors declare that the research was conducted in the absence of any commercial or financial relationships that could be construed as a potential conflict of interest.

## Publisher’s Note

All claims expressed in this article are solely those of the authors and do not necessarily represent those of their affiliated organizations, or those of the publisher, the editors and the reviewers. Any product that may be evaluated in this article, or claim that may be made by its manufacturer, is not guaranteed or endorsed by the publisher.
