# Supplementary material for: Skeletal Muscle Adaptations and Performance Outcomes Following a Step and Exponential Taper in Strength Athletes
Source: Front Physiol. 2021 Oct 21;12:735932. doi: 10.3389/fphys.2021.735932 (PMC8582352; doi:10.3389/fphys.2021.735932)
Supplement: Supplementary file 1 [file Table_1.docx]

**Supplementary Table 1.** Forward and reverse primer sequences for analyzed mRNAs.

| **Gene** | **Sequence** |
| --- | --- |
| Target Gene | |
| PAX 7 (Forward) | CCTTTGGAAGTGTCCACCCC |
| PAX 7 (Reverse) | TCGCCCATTGATGAAGACCC |
| Myostatin (Forward) | CTACAACGGAAACAATCATTACCA |
| Myostatin (Reverse) | GTTTCAGAGATCGGATTCCAGTAT |
| MyoD (Forward) | CGGCATGATGGACTACAGCG |
| MyoD (Reverse) | CAGGCAGTCTAGGCTCGAC |
| MyoG (Forward) | GGCCAAACTTTTGCAGTGAATATT |
| MyoG (Reverse) | TCGGATGGCAGCTTTACAAACAAC |
| Sox6 (Forward) | GCAAGAACAGATTGCGAGAC |
| Sox6 (Reverse) | AATTGGGATCATGAGCGGAGG |
| MYH7 (Forward) | AGAAGATGTGCCGGACCTTG |
| MYH7 (Reverse) | GACAGCTCACCATTCTCGGT |
| MYH2 (Forward) | GCTTTAAAAAGCTCCAAGAACTGTC |
| MYH2 (Reverse) | ACTTTCGGAGGAAAGGAGCAG |
| MYH1 (Forward) | CCAGACTGTGTCTGCTCTCT |
| MYH1 (Reverse) | CCATGGCACCAGGAGTTTTA |
| Housingkeeping References | |
| EMC7 (Forward) | GGGCTGGACAGACTTTCTAATG |
| EMC7 (Reverse) | CTCCATTTCCCGTCTCATGTCAG |
| VCP (Forward) | AAACTCATGGCGAGGTGGAG |
| VCP (Reverse) | TGTCAAAGCGACCAAATCGC |
| CHMP2A (Forward) | CGCTATGTGCGCAAGTTTGT |
| CHMP2A (Reverse) | GGGGCAACTTCAGCTGTCTG |
| C1orf43 (Forward) | CTATGGGACAGGGGTCTTTGG |
| C1orf43 (Reverse) | TTTGGCTGCTGACTGGTGAT |
