# Supplementary material for: Skeletal Muscle Adaptations and Performance Outcomes Following a Step and Exponential Taper in Strength Athletes
Source: Front Physiol. 2021 Oct 21;12:735932. doi: 10.3389/fphys.2021.735932 (PMC8582352; doi:10.3389/fphys.2021.735932)
Supplement: Supplementary file 2 [file Table_2.docx]

**Supplementary Table 2.** Catalogue numbers for the miRNAs analyzed and housekeeping references with Thermo Fisher Scientific independent miR assay IDs.

|  | |
| --- | --- |
| **miR** | **ID Number** |
| Target | |
| *miR-23a-3p* | 478532_mir |
| *miR-451a* | 477968_mir |
| *miR-486-5p* | 478128_mir |
| *miR-133a-3p* | 478511_mir |
| *miR-206* | 477968_mir |
| *miR-499a-3p* | 478948_mir |
|  | |
| Housingkeeping References | |
| *miR-186-5p* | 477940_mir |
| *miR-320a* | 478594_mir |
| *miR-361-5p* | 478056_mir |
